# Patterns of occurrence and implications of neratinib-associated diarrhea in patients with HER2-positive breast cancer: analyses from the randomized phase III ExteNET trial

**DOI:** 10.1186/s13058-019-1112-5

**Published:** 2019-02-27

**Authors:** Joanne Mortimer, Jack Di Palma, Kendra Schmid, Yining Ye, Mohammad Jahanzeb

**Affiliations:** 1 City of Hope Comprehensive Cancer Center, Duarte, CA 91010 USA; 2 University of South Alabama College of Medicine, Mobile, AL USA; 3 College of Public Health, University of Nebraska Medical Center, Omaha, NE USA; 4 Puma Biotechnology Inc., South San Francisco, CA USA; 5 Sylvester Comprehensive Cancer Center, University of Miami, Miller School of Medicine, Deerfield Beach, FL USA; 6 Present address: QED Therapeutics, San Francisco, CA USA

**Keywords:** Early-stage breast cancer, HER2 positive, Neratinib, Pan-HER inhibition, Diarrhea, Health-related quality of life, Patient-reported outcomes

## Abstract

**Background:**

We characterized patterns of occurrence and the impact of neratinib-associated diarrhea in the absence of protocol-directed antidiarrheal prophylaxis or a formal diarrhea management plan using data from Extended Adjuvant Treatment of Breast Cancer with Neratinib (ExteNET).

**Methods:**

ExteNET is a multicenter, double-blind, placebo-controlled, randomized phase III trial involving community-based and academic institutions in 40 countries. Women with HER2-positive early-stage breast cancer with prior standard primary therapy and trastuzumab-based (neo)adjuvant therapy were randomized to neratinib 240 mg/day or placebo for 12 months. Safety, a secondary outcome, was assessed using the National Cancer Institute Common Terminology Criteria version 3.0. Health-related quality of life by diarrhea grade was assessed using Functional Assessment of Cancer Therapy-Breast (FACT-B).

**Results:**

Two thousand eight hundred sixteen women (1408 per group) were safety-evaluable. Grade 3 and 4 diarrhea occurred in 561 (39.8%) and 1 (0.1%) patients with neratinib versus 23 (1.6%) and 0 patients with placebo, respectively. In the neratinib group, 28.6% of patients had grade 3 events during month 1 decreasing to ≤ 6% after month 3. The median cumulative duration of grade 3/4 diarrhea with neratinib was 5 days (interquartile range, 2–9). Serious diarrheal events (*n* = 22, 1.6%) and diarrheal events requiring hospitalization (*n* = 20, 1.4%) were rare with neratinib. Changes in FACT-B total score by diarrhea grade in the neratinib group did not meet the threshold for clinically important differences.

**Conclusions:**

In the absence of antidiarrheal prophylaxis, neratinib-related diarrhea is short-lived and not associated with complications or long-term sequelae. This suggests that targeted preventive management with antidiarrheal prophylaxis early during neratinib treatment is appropriate.

**Trial registration:**

ClinicalTrials.gov NCT00878709. Registered 9 April 2009.

**Electronic supplementary material:**

The online version of this article (10.1186/s13058-019-1112-5) contains supplementary material, which is available to authorized users.

## Background

Diarrhea is a well-recognized side effect of chemotherapeutic and targeted regimens used to treat breast cancer, including pertuzumab-based regimens [1, 2], docetaxel plus capecitabine [3], and capecitabine plus lapatinib [4]. Despite the frequency of treatment-induced diarrhea, few clinically relevant studies have attempted to understand diarrhea; management guidelines for cancer treatment-related diarrhea remain consensus-based rather than evidence-based [5].

Neratinib (Nerlynx®; Puma Biotechnology Inc., Los Angeles, CA) is a potent, irreversible tyrosine kinase inhibitor of human epidermal growth factor receptor (HER or ERBB) 1, 2, and 4 [6]. Single-agent neratinib is effective in the treatment of metastatic HER2-positive breast cancer [7–9], including trastuzumab-naïve and trastuzumab-pretreated patients [8], and *HER2* mutation-positive breast cancer [10]. In the first-line treatment of metastatic HER2-positive breast cancer, neratinib plus paclitaxel had similar efficacy to trastuzumab plus paclitaxel, but significantly delayed the onset and reduced the frequency of central nervous system progression [11].

Neratinib is approved in the USA for the extended adjuvant treatment of early-stage HER2-positive breast cancer after standard trastuzumab-based adjuvant therapy based on findings from the ExteNET (Extended Adjuvant Treatment of Breast Cancer with Neratinib) trial. The primary analysis showed that neratinib significantly improved 2-year invasive disease-free survival (iDFS) versus placebo (stratified hazard ratio [HR] 0.67, 95% confidence interval [CI] 0.50–0.91; *P =* 0*.*0091) [12]. The significant benefits of neratinib on iDFS were maintained after 5 years of follow-up [13]. Grade 3 adverse events with neratinib were reported infrequently (< 5% each), with the exception of diarrhea which was common (grade 3, 40%; grade 4, 0.1%) [12].

In early neratinib trials—including ExteNET—no formal management plan existed for neratinib-associated diarrhea, and symptoms were managed reactively. Later trials included loperamide prophylaxis for the first 1–2 cycles to help manage neratinib-associated diarrhea [14]. The ExteNET Independent Data Monitoring Committee (IDMC) believes that safety data from ExteNET provide a unique opportunity to study the clinical pattern of neratinib-associated diarrhea and its consequences in a large patient population. The IDMC herein reports analyses on diarrhea and its sequelae in the ExteNET trial.

## Methods

### Study design

ExteNET is a double-blind, placebo-controlled, randomized phase III trial performed in community-based and academic centers in Europe, Asia, Australia, New Zealand, and North and South America (Clinicaltrials.gov identifier: NCT00878709). The study design evolved through three global protocol amendments as described previously [12]. The final study design comprised three parts: part A, primary efficacy and safety analysis at 2 years [12]; part B, sensitivity analysis of efficacy endpoints at 5 years [13]; and part C, analysis of overall survival after 248 events for which patient follow-up is ongoing. Randomization type, methods used to generate the randomization sequence, stratification factors, and masking details were previously described [12].

The study was performed in accordance with the 2008 Declaration of Helsinki; the protocol was approved by institutional review boards/independent ethics committees at each site. Data were reviewed by the IDMC at least twice yearly.

### Patients

Women with histologically confirmed invasive breast cancer with *HER2* gene amplification or HER2 receptor overexpression were eligible. Patients were required to have stage 1–3c disease (amended to 2–3c disease in February 2010), prior surgery and trastuzumab-based (neo)adjuvant therapy completed ≤ 2 years (amended to 1 year in February 2010) before randomization, and adequate organ function. Concurrent adjuvant endocrine therapy for hormone receptor-positive disease was permitted; concurrent chemotherapy, radiotherapy, immunotherapy, or biotherapy for breast cancer were not. Patients provided written informed consent before study participation.

### Procedures

Patients were randomly assigned (1:1) to receive oral neratinib 240 mg once daily continuously or matching placebo for 1 year or until disease recurrence/new breast cancer, intolerable adverse events, or consent withdrawal. Neratinib dose reductions (to 200, 160, and 120 mg/day) were permitted for toxicity. Treatment was stopped if the 120-mg dose level was not tolerated or if treatment was interrupted for more than 3 weeks. There was no formal management plan or primary prophylaxis for diarrhea, but investigators were advised to treat grade 1 or higher diarrhea with loperamide according to the following schedule: loperamide 4 mg at first onset of diarrhea and then 2 mg every 4 h or after each loose stool until diarrhea free for at least 12 h. Dose holds were recommended for grade 2 or 3 diarrhea not improving to grade ≤ 1 within 24 to 48 h and for recurrent grade 3 events, and dose reductions were recommended for recurrent grade 2 diarrhea and grade 3 events.

### Outcomes

Safety was a secondary outcome. Adverse events were monitored until 28 days after the last dose of study drug and graded according to National Cancer Institute Common Terminology Criteria, version 3.0; the definitions assigned to each grade of diarrhea according to these criteria are presented in Additional file 1: Table S1. Serious adverse events were defined as any event that resulted in death, was life-threatening, required inpatient hospitalization or prolongation of existing hospitalization, resulted in persistent or significant disability/incapacity (substantial disruption of the ability to conduct normal life functions), or resulted in congenital anomaly/birth defect.

Health-related quality of life (HRQoL) was assessed using Functional Assessment of Cancer Therapy-Breast (FACT-B) [15], version 4. Patients completed questionnaires at baseline; months 1, 3, 6, and 9; and end of treatment. Minimally important differences (MIDs; 7–8 points) reported in the literature [16] were used to interpret changes in FACT-B total score.

### Statistical analysis

Adverse events were summarized descriptively by treatment arm for the safety population, defined as all patients who received ≥ 1 dose of study drug. Summaries focused on treatment-emergent adverse events, defined as any adverse event occurring or worsening on or after the first dose of study drug and ≤ 28 days after the last dose (cut-off for safety analyses: July 2014).

Additional exploratory analyses included ordinal logistic regression to investigate potential predictors of diarrhea, expressed as odds ratios (ORs; with 95% CIs) of having higher grade (grade 2, 3 or 4) versus lower grade or no diarrhea (grade 0 or 1). Covariates included time from last trastuzumab dose to randomization, age, baseline Eastern Cooperative Oncology Group performance status (0, 1), race (white, Asian, other), concomitant endocrine therapy (anti-estrogen and aromatase inhibitor, aromatase inhibitor only, anti-estrogen only, other), adjuvant chemotherapy regimen (anthracycline plus taxane, anthracycline only, neither anthracycline nor taxane, taxane only), concomitant proton pump inhibitor (yes, no), baseline body mass index, and baseline renal status (normal, mild, moderate, severe). Kaplan-Meier methods were used to investigate the impact of neratinib-associated diarrhea on outcomes by performing an analysis of 2-year iDFS, the primary efficacy endpoint, by maximal diarrhea grade in the first 7 days. Descriptive analyses of FACT-B total scores over time by maximum diarrhea grade (no or grade 1 diarrhea, grade 2 diarrhea, grade 3 diarrhea) were performed in the safety population with no imputation for missing values; a sensitivity analysis was performed using last observation carried forward (LOCF) as an imputation method for missing values.

## Results

Overall, 2840 patients were enrolled between July 9, 2009, and October 24, 2011. All were included in the intention-to-treat population (1420 per group); 2816 (1408 per group) received ≥ 1 dose of study treatment and were included in the safety population (Additional file 1: Figure S1). Baseline characteristics are presented in Additional file 1: Table S2. The median interval from last trastuzumab dose to randomization was 4.4 months for neratinib and 4.6 months for placebo. The median duration of treatment was 11.6 (interquartile range [IQR], 2.5–11.9) months for neratinib and 11.8 (IQR, 11.5–12.0) for placebo; reasons for discontinuing treatment are shown in Additional file 1: Figure S1.

### Patterns of occurrence and consequences of diarrhea

Grade 1, 2, 3, and 4 diarrhea occurred in 323 (22.9%), 458 (32.5%), 561 (39.8%), and 1 (0.1%) patients in the neratinib group and 382 (27.1%), 94 (6.7%), 23 (1.6%), and 0 patients in the placebo group, respectively (Table 1). Serious diarrhea events were rare in both treatment groups (neratinib, 1.6%; placebo, 0.1%).

**Table 1:** Summary of treatment-emergent diarrhea (safety population)

Variable	Neratinib (*N* = 1408)	Placebo (*N* = 1408)
Any treatment-emergent diarrhea, *N* (%)	1343 (95.4)	499 (35.4)
Drug-related diarrhea, *N* (%)	1330 (94.5)	411 (29.2)
Serious events,^a^ *N* (%)	22 (1.6)	1 (0.1)
Maximum toxicity, *N* (%)		
Grade 1	323 (22.9)	382 (27.1)
Grade 2	458 (32.5)	94 (6.7)
Grade 3	561 (39.8)	23 (1.6)
Grade 4	1 (0.1)	0
Outcome of last diarrhea episode, *N* (%)		
Resolved	1276 (90.6)	483 (34.3)
Persisted^b^	67 (4.8)	16 (1.1)
Median (IQR) time to first onset of diarrhea, days		
Any grade	2 (2–4)	18 (4–82)
Grade ≥ 2	5 (2–15)	90 (17–189)
Grade ≥ 3	8 (4–33)	124 (21–257)
Median (IQR) duration of diarrhea per event, days		
Any grade	2 (1–3)	2 (1–3)
Grade ≥ 2	1 (1–2)	2 (1–2)
Grade ≥ 3	2 (1–3)	2 (1–4)
Median (IQR) cumulative duration of diarrhea per patient,^c^ days		
Any grade	59 (14–164)	6 (2–34)
Grade ≥ 2	10 (5–27)	3 (2–7)
Grade ≥ 3	5 (2–9)	2 (1–5)
Median (IQR) episodes per patient, *n*		
Any grade	8 (3–27)	2 (1–6)
Grade ≥ 2	3 (1–8)	1 (1–2)
Grade ≥ 3	2 (1–3)	1 (1–1)

*IQR* interquartile range, *SD* standard deviation

^a^Defined as any event that resulted in death, was life-threatening, required inpatient hospitalization or prolongation of existing hospitalization, resulted in persistent or significant disability/incapacity (substantial disruption of the ability to conduct normal life functions), or resulted in congenital anomaly/birth defect

^b^Persisted beyond 28 days after the last dose of study drug

^c^Defined as the sum of the durations of all episodes of diarrhea of the grade specified

Neratinib-associated diarrhea occurred early during treatment regardless of grade. The median time to onset for grade ≥ 3 events with neratinib was 8 (IQR, 4–33) days, and for any-grade diarrhea was 2 (IQR 2–4) days (Table 1).

With neratinib, most diarrhea occurred during the first month; the frequency of grade ≥ 3 events decreased in subsequent months. The incidence of grade 3 diarrhea decreased from 28.6% (month 1) to 11.2% (month 2), and ≤ 6% after month 3; grade 2 diarrhea decreased steadily from 30.1% in month 1 to 11.8% in month 12. Grade 1 diarrhea did not change appreciably over time, affecting about 30% of patients intermittently throughout the treatment period.

Neratinib-associated diarrhea events were generally of short duration, lasting a median of 1–2 days per event regardless of severity (Table 1). The median cumulative duration of grade ≥ 3 diarrhea per patient (defined as the sum of the durations of all episodes of diarrhea of that grade) was 5 (IQR 2–9) days versus 2 (IQR 1–5) days with placebo (Table 1). The median cumulative duration of any grade events per patient was 59 (IQR 14–164) days with neratinib versus 6 (IQR 2–34) days with placebo (Table 1). Persistent diarrhea lasting > 28 days after the last dose of study treatment was uncommon (neratinib 4.8%; placebo 1.1%) (Table 1); most of these events were grade 1, with persistent grade 3 events reported in 2 patients in the neratinib group.

Twenty patients (1.4%) in the neratinib group were hospitalized because of diarrhea compared with 1 (0.1%) patient in the placebo group, and 237 (16.8%) and 3 (0.2%) patients, respectively, discontinued study treatment because of diarrhea (Table 2). Diarrhea-related dose reductions were more common with neratinib than placebo (Table 2). Most neratinib-treated patients with dose modifications required only one dose reduction or dose hold to manage their diarrhea (Table 2). Dose holds, dose reductions and treatment discontinuations because of neratinib-associated diarrhea generally occurred within the first 3 weeks of treatment with median times to occurrence of 8 (IQR 3–29) days, 19 (IQR 6–53) days, and 20 (IQR 9–56) days, respectively (Table 2). Neratinib-treated patients with grade 2 or 3 diarrhea required more dose reductions and dose holds and had a lower relative dose intensity than patients with grade 1 or no diarrhea (Additional file 1: Table S3).

**Table 2:** Actions required because of treatment-emergent diarrhea (safety population)

Action	Neratinib (*N* = 1408)	Placebo (*N* = 1408)
Hospitalization, *N* (%)	20 (1.4)	1 (0.1)
Withdrawn from study, *N* (%)	23 (1.6)	0
Discontinued study drug, *N* (%)	237 (16.8)	3 (0.2)
Grade 1	60 (4.3)	0
Grade 2	71 (5.0)	2 (0.1)
Grade 3	106 (7.5)	1 (0.1)
Median (IQR) time to discontinuation, days	20 (9–56)	241 (147–305)
Dose reduction, *N* (%)	372 (26.4)	8 (0.6)
Once	239 (17.0)	6 (0.4)
Twice	96 (6.8)	1 (0.1)
Three or more times^a^	37 (2.6)	1 (0.1)
Median (IQR) time to dose reduction, days	19 (6–53)	99 (18–252)
Dose hold, *N* (%)	477 (33.9)	26 (1.8)
Once	263 (18.7)	22 (1.6)
Twice	97 (6.9)	2 (0.1)
Three or more times	117 (8.3)	2 (0.1)
Median (IQR) time to dose hold, days	8 (3–29)	64 (19–239)

*IQR* interquartile range

^a^Also included cases of dosing errors and dosing noncompliance

The occurrence of diarrhea with neratinib did not appear to affect clinical outcomes: patients with diarrhea during the first 7 days of initiating neratinib appeared to have similar iDFS Kaplan-Meier curves over 2 years as those reporting no diarrhea within the first 7 days (Additional file 1: Figure S2).

### Predictors of diarrhea

Apart from study treatment (neratinib), race was the only baseline factor significantly associated with the occurrence of higher-grade diarrhea (Additional file 1: Table S4). Asian patients were significantly more likely to experience higher-grade diarrhea versus white patients (OR 1.6; 95% CI 1.3–2.0; *P* < 0.0001), and patients of other races were significantly less likely to experience higher-grade diarrhea versus white patients (OR 0.6, 95% CI 0.4–0.8; *P* < 0.0001).

### Complications of diarrhea

**Fig. 1:**
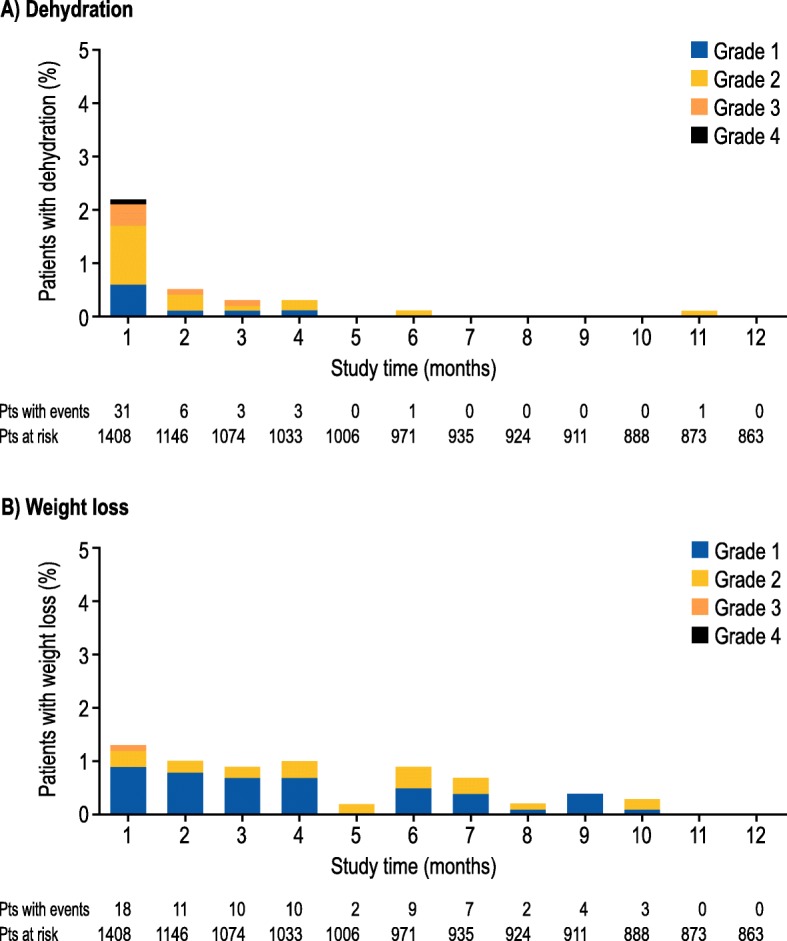
Incidence of treatment-emergent **a** dehydration and **b** weight loss by grade over time in the neratinib group

Adverse events potentially associated with complicated cases of diarrhea are shown in Table 3. Grade 3 events with neratinib were uncommon (< 4%), and grade 4 events were rare (< 1%). The incidence of dehydration in the neratinib group (Fig. 1a) decreased over time, with the overall incidence of dehydration showing a marked drop-off as for grade 3 diarrhea. The incidence of weight loss in the neratinib group (Fig. 1b) appeared to show little correlation with the occurrence of diarrhea, and the number of events, predominantly grade 1, was consistently small throughout the study.

**Table 3:** Clinical adverse events potentially associated with complicated diarrhea (safety population)

Adverse event, *N* (%)	Neratinib (*N* = 1408)				Placebo (*N* = 1408)			
	Grade 1	Grade 2	Grade 3	Grade 4	Grade 1	Grade 2	Grade 3	Grade 4
Nausea	439 (31.2)	140 (9.9)	26 (1.8)	0	269 (19.1)	32 (2.3)	2 (0.1)	0
Vomiting^a^	221 (15.7)	101 (7.2)	47 (3.3)	0	86 (6.1)	21 (1.5)	5 (0.4)	0
Fatigue^a^	256 (18.2)	103 (7.3)	23 (1.6)	0	214 (15.2)	62 (4.4)	6 (0.4)	0
Abdominal pain^a^	232 (16.5)	82 (5.8)	24 (1.7)	0	119 (8.5)	22 (1.6)	3 (0.2)	0
Abdominal pain upper	160 (11.4)	41 (2.9)	11 (0.8)	0	85 (6.0)	8 (0.6)	3 (0.2)	0
Decreased appetite^a^	136 (9.7)	30 (2.1)	3 (0.2)	0	34 (2.4)	6 (0.4)	0	0
Weight decreased	44 (3.1)	23 (1.6)	1 (0.1)	0	5 (0.4)	2 (0.1)	0	0
Pyrexia	66 (4.7)	13 (0.9)	0	0	47 (3.3)	8 (0.6)	0	0
Dehydration	12 (0.9)	26 (1.8)	12 (0.9)	1 (0.1)	2 (0.1)	2 (0.1)	1 (0.1)	0
Neutropenia	5 (0.4)	13 (0.9)	0	1 (0.1)	7 (0.5)	4 (0.3)	2 (0.1)	0
Nephrotoxicity	13 (0.9)	5 (0.4)	7 (0.5)	1 (0.1)	4 (0.3)	2 (0.1)	0	0
Blood creatinine increased	9 (0.6)	3 (0.2)	1 (0.1)	1 (0.1)	3 (0.2)	1 (0.1)	0	0
Blood urea increased	1 (0.1)	1 (0.1)	1 (0.1)	0	0	0	0	0
Glomerular filtration rate decreased	1 (0.1)	0	0	0	0	0	0	0
Protein urine present	0	0	0	0	0	1 (0.1)	0	0
Renal failure	0	1 (0.1)	3 (0.2)	0	0	0	0	0
Acute renal failure	1 (0.1)	1 (0.1)	3 (0.2)	0	0	0	0	0
Renal function test abnormal	1 (0.1)	0	0	0	0	0	0	0
Renal impairment	0	0	0	0	1 (0.1)	0	0	0
Hypokalemia	17 (1.2)	0	4 (0.3)	1 (0.1)	11 (0.8)	0	5 (0.4)	0
Hyponatremia	4 (0.3)	0	5 (0.4)	0	2 (0.1)	0	5 (0.4)	2 (0.1)

^a^Missing grades: vomiting (placebo, *n* = 1), fatigue (placebo, *n* = 1), abdominal pain (neratinib, *n* = 2), and decreased appetite (neratinib, *n* = 1)

Blood chemistry results outside the normal range are summarized in Additional file 1: Table S5. The only blood chemistry result differing appreciably between groups was creatinine: 12.5% of neratinib-treated and 9.2% of placebo-treated patients had elevated blood creatinine levels. The proportion of patients with electrolyte results outside the normal range was similar in both treatment groups, except for hypocalcemia, which was slightly more common with neratinib (48.9% vs 42.9% with placebo).

### Effects of diarrhea on health-related quality of life

**Fig. 2:**
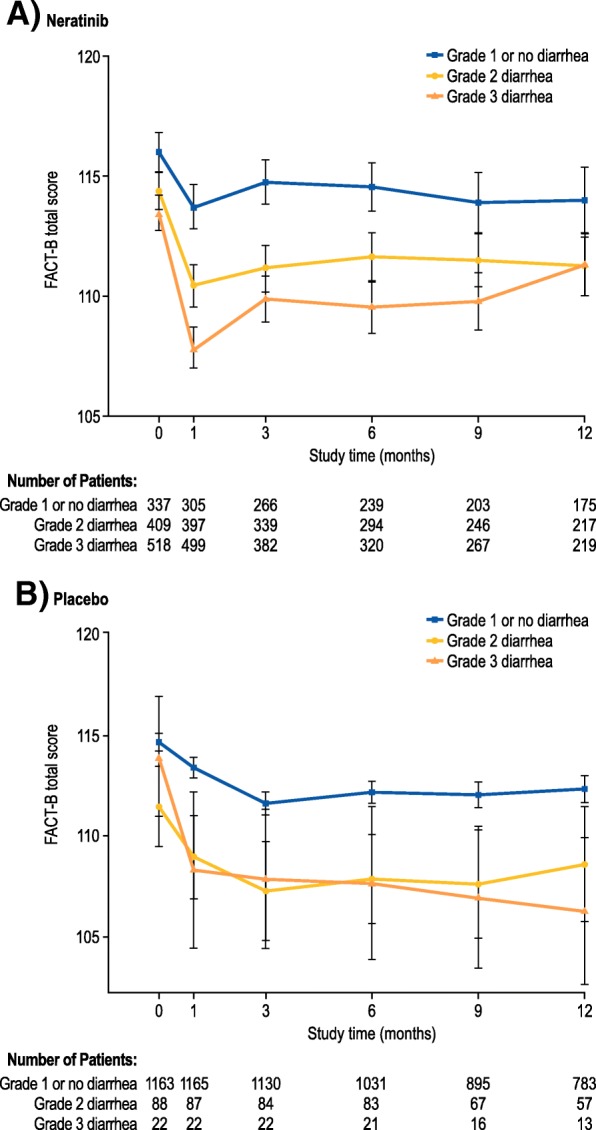
Mean (± standard deviation) Functional Assessment of Cancer Therapy-Breast (FACT-B) total scores over time by diarrhea grade for **a** neratinib and **b** placebo. For FACT-B: minimally important difference, 7–8 points [16]. A higher score indicates a better quality of life

In the safety population, 1264 neratinib-treated patients and 1273 placebo-treated patients completed ≥ 1 HRQoL assessment. Figure 2 a and b show mean FACT-B total scores by diarrhea grade over time without imputation for missing values. In the neratinib group, maximum decreases in FACT-B total scores were short-lived and observed at month 1; changes were less than the MID (i.e., 7–8 points). Thereafter, FACT-B total scores showed a gradual improvement regardless of diarrhea grade. In the placebo group, the maximum decreases in FACT-B total scores by diarrhea grade occurred at month 3 or later and were similar to the maximum decreases observed with neratinib. Similar changes in FACT-B total scores were observed in a sensitivity analysis using LOCF (Additional file 1: Figure S3).

## Discussion

The ExteNET trial has permitted a detailed investigation of patterns of occurrence and impact of diarrhea associated with neratinib monotherapy in the absence of protocol-directed antidiarrheal prophylaxis or a formal diarrhea management plan. The observed rate of grade 3 neratinib-associated diarrhea was high, but had a distinct clinical course. Diarrhea tended to occur early (median time to onset 2 days for all-grade events), and most grade ≥ 3 events occurred within the first month, with a marked drop-off in frequency thereafter. The cumulative duration of grade ≥ 3 diarrhea with neratinib was short (median 5 days). Importantly, neratinib-associated diarrhea did not appear to adversely affect clinical outcomes.

In ExteNET, diarrhea was treated with loperamide when symptoms became apparent, and neratinib dose modifications were recommended in cases of grade 2 or 3 diarrhea. A structured regimen of loperamide prophylaxis during the first 1–2 cycles of neratinib therapy has since been shown to reduce the incidence, severity, and duration of diarrheal events in the phase II CONTROL study [17] compared with those observed in ExteNET and is now recommended for the management of neratinib-associated diarrhea. In the CONTROL study [17], no diarrhea was reported in 20% of patients with loperamide prophylaxis versus 5% in ExteNET, and grade 2 or 3 diarrhea occurred in 55% of patients versus 72% in ExteNET; the median cumulative duration of any-grade diarrhea was 14 days in CONTROL versus 59 days in ExteNET. Efforts to further improve the effectiveness of loperamide prophylaxis are continuing through the addition of agents targeting inflammation (budesonide) and bile acid malabsorption (colestipol). Interim data suggest grade 3 diarrhea rates of 27% with loperamide plus budesonide and 11% with loperamide plus colestipol vs 40% in ExteNET, and treatment discontinuation rates due to diarrhea of 11% and 2% vs 17%, respectively [17].

Even without antidiarrheal prophylaxis in ExteNET, neratinib-related diarrhea does not appear to follow a complicated course and is associated with little morbidity. Hospital admissions because of diarrhea (1.4%) and serious diarrheal events (1.6%) with neratinib were rare. A review of unsolicited post-treatment serious adverse events from ExteNET showed no serious late-term consequences from neratinib-associated diarrhea (e.g., renal insufficiency or chronic intestinal disease) [13]. Further, many of the signs and symptoms suggestive of complicated diarrhea (i.e., fever, sepsis, neutropenia, bleeding, and dehydration) [18] were no more frequent with neratinib than placebo, and loss of fluids and changes in electrolytes, which can occur with persistent or severe diarrhea, also generally occurred at similar rates in both groups.

ExteNET included a detailed evaluation of patient-reported outcomes using validated HRQoL questionnaires. According to these assessments, HRQoL impairment with neratinib was maximal at month 1, but returned toward baseline thereafter. These changes were likely attributable to the occurrence of diarrhea during month 1, although they were less than the MID threshold regardless of diarrhea grade, and so are not considered to be clinically meaningful. The FACT-B questionnaire does not include any diarrhea-specific questions and may not be sensitive enough to capture meaningful variations, although a clear incremental decrease in FACT-B total score was evident with increasing diarrhea severity (Fig. 2), suggestive of some sensitivity to this adverse event. Full HRQoL findings from the ExteNET study are reported separately [19].

The pattern of diarrheal events with neratinib appears to differ from other regimens. For pertuzumab, the time to the first onset of diarrhea occurs slightly later than that with neratinib (median 5–8 days [1] versus 2 days with neratinib); no data on duration of pertuzumab-associated diarrhea are published [1]. For lapatinib, most first diarrhea events occur early (45% of patients within 6 days) [20] as for neratinib, but the median duration of each event appears to be longer (5–6 days [20] vs 2 days with neratinib). Limited data regarding chemotherapy-associated diarrhea suggest a different course of events [21]. HRQoL impairment with chemotherapy-induced diarrhea also appears to be greater than with neratinib [22, 23].

Race was a prognostic factor for the occurrence of higher-grade diarrhea. Lower bodyweight among Asian patients may have contributed to this outcome, although body mass index was not a significant predictor of diarrhea in the same analysis. Another possible explanation is that Asian patients in ExteNET were less likely to discontinue study treatment because of adverse events compared with the overall safety population [24]. This led to higher rates of some adverse events, including diarrhea, among Asian patients, although importantly efficacy outcomes among Asians were unaffected [24].

Whereas safety was a predefined secondary endpoint, some of the analyses described herein were exploratory. ExteNET included almost 3000 women from Europe, Asia, Americas, and Australasia; therefore, these findings are likely to be broadly generalizable to other populations with HER2-positive breast cancer.

## Conclusions

In conclusion, neratinib-associated diarrhea has a distinct and predictable clinical course. Grade 3 events are generally short-lived and occur within the first month of treatment, permitting targeted preventive management with antidiarrheal prophylaxis early in the treatment course. Serious diarrheal events and hospital admissions because of diarrhea were rare, suggesting that most cases do not follow a complicated course.

## Additional file

Additional file 1: **Table S1.** Diarrhea grading (National Cancer Institute Common Terminology Criteria, version 3.0). **Table S2.** Baseline characteristics of patients (intention-to-treat population)**.** **Table S3.** Drug exposure by diarrhea grade (safety population). **Table S4.** Predictors of diarrhea. **Table S5.** Blood chemistry results outside of normal range (safety population). **Figure S1.** ExteNET CONSORT flowchart. **Figure S2.** Kaplan-Meier curves of invasive disease-free survival by worst grade of diarrhea experienced by patients in the neratinib group in the first 7 days (intention-to-treat population with a treatment duration > 1 month). **Figure S3.** Mean (± standard deviation) Functional Assessment of Cancer Therapy – Breast (FACT-B) total scores over time by diarrhea grade for (A) neratinib and (B) placebo with last observation carried forward. (PDF 305 kb)

## Abbreviations

CI: Confidence interval
ExteNET: Extended Adjuvant Treatment of Breast Cancer with Neratinib
FACT-B: Functional Assessment of Cancer Therapy-Breast
HER: Human epidermal growth factor receptor
HR: Hazard ratio
HRQoL: Health-related quality of life
iDFS: Invasive disease-free survival
IDMC: Independent Data Monitoring Committee
IQR: Interquartile range
LOCF: Last observation carried forward
MID: Minimally important difference
OR: Odds ratio
