# Supplementary material for: Patterns of occurrence and implications of neratinib-associated diarrhea in patients with HER2-positive breast cancer: analyses from the randomized phase III ExteNET trial
Source: Breast Cancer Res. 2019 Feb 27;21:32. doi: 10.1186/s13058-019-1112-5 (PMC6391844; doi:10.1186/s13058-019-1112-5)
Supplement: Supplementary file 1 — Table S1. Diarrhea grading (National Cancer Institute Common Terminology Criteria, version 3.0). Table S2. Baseline characteristics of patients (intention-to-treat population). Table S3. Drug exposure by diarrhea grade (safety population). Table S4. Predictors of diarrhea. Table S5. Blood chemistry results outside of normal range (safety population). Figure S1. ExteNET CONSORT flowchart. Figure S2. Kaplan-Meier curves of invasive disease-free survival by worst grade of diarrhea experienced by patients in the neratinib group in the first 7 days (intention-to-treat population with a treatment duration > 1 month). Figure S3. Mean (± standard deviation) Functional Assessment of Cancer Therapy – Breast (FACT-B) total scores over time by diarrhea grade for (A) neratinib and (B) placebo with last observation carried forward. (PDF 305 kb) [file 13058_2019_1112_MOESM1_ESM.pdf]

Electronic Supplementary Material

Title: Patterns of occurrence and implications of neratinib-associated diarrhea in patients with HER2-positive breast cancer: analyses from the randomized phase 3 ExteNET trial

Journal: *Breast Cancer Research and Treatment*

Authors: Joanne Mortimer, Jack Di Palma, Kendra Schmid, Yining Ye, Mohammad Jahanzeb

Corresponding author: Prof. Joanne Mortimer

City of Hope Comprehensive Cancer Center

1500 East Duarte Road, Duarte

CA 91010

USA

E-mail: JMortimer@coh.org

Supplementary Table 1. Diarrhea grading (National Cancer Institute Common Terminology Criteria, version 3.0)

Grade	Description
1	Increase of <4 stools per day or mild increase in ostomy output compared with baseline
2	Increase of 4–6 stools per day or moderate increase in ostomy output compared with baseline, intravenous fluids <24 hours, and no interference with activities of daily living
3	Increase of ≥7 stools per day or severe increase in ostomy output compared with baseline, incontinence, intravenous fluids ≥24 hours, hospitalization, and interference with activities of daily living
4	Life-threatening consequences (e.g. hemodynamic collapse) or death

Supplementary Table 2. Baseline characteristics of patients (intention-to-treat population)[a]

Characteristic	Neratinib (*N* = 1420)	Placebo (*N* = 1420)
Region		
North America	519 (37)	477 (34)
Western Europe, Australia, New Zealand and South Africa	487 (34)	532 (38)
Asia Pacific, East Europe and South America	414 (29)	411 (29)
Race		
White	1165 (82)	1135 (80)
Black	27 (2)	47 (3)
Asian	188 (13)	197 (14)
Other	40 (3)	41 (3)
Age at randomization, years		
<35	46 (3)	55 (4)
35–49	523 (37)	515 (36)
50–59	497 (35)	488 (34)
≥60	354 (25)	362 (26)
Age, years		
Median (range)	52.0 (25.0–83.0)	52.0 (23.0–82.0)
Menopausal status at diagnosis		
Premenopausal	663 (47)	664 (47)
Postmenopausal	757 (53)	756 (53)
Nodal status[b]		
Negative	335 (24)	336 (24)
1–3 positive nodes	664 (47)	664 (47)
≥4 positive nodes	421 (30)	420 (30)
Hormone receptor status[b]		
Positive (ER and/or PR positive)	816 (58)	815 (57)
Negative (ER and PR negative)	604 (43)	605 (43)
Prior trastuzumab regimen[b]		
Concurrent	884 (62)	886 (62)
Sequential	536 (38)	534 (38)
T category		
T1	440 (31)	459 (32)
T2	585 (41)	555 (39)
T3 and above	144 (10)	117 (8)
Unknown	250 (18)	288 (20)
Missing	1 (<1)	1 (<1)
Histologic grade of tumor		
Undifferentiated/poorly differentiated	670 (47)	689 (49)

Moderately differentiated	461 (33)	416 (29)
Well differentiated	76 (5)	65 (5)
Unknown	213 (15)	241 (17)
Prior surgery		
Lumpectomy only	468 (33)	511 (36)
Mastectomy	951 (67)	908 (64)
Missing	1 (<1)	1 (<1)
Prior radiotherapy		
Yes	1130 (80)	1150 (81)
No	290 (20)	270 (19)
Prior (neo)adjuvant therapy[c]		
Yes	1420 (100)	1420 (100)
Trastuzumab	1420 (100)	1420 (100)
Anthracycline only	136 (10)	135 (10)
Anthracycline plus taxane	962 (68)	965 (68)
Taxane only	318 (22)	316 (22)
Non-anthracycline or taxane	4 (<1)	4 (<1)
Duration of prior adjuvant trastuzumab therapy, months	*N* = 1413	*N* = 1416
Median (IQR)	11.5 (10.9-11.9)	11.4 (10.8-11.9)
Time since last dose of trastuzumab to randomization, months		
Median (IQR)	4.4 (1.6-10.4)	4.6 (1.5-10.8)
Concomitant endocrine therapy for patients with hormone-positive disease[d]		
No	56 (7)	51 (6)
Yes	760 (93)	764 (94)
Anti-estrogen only	375 (46)	347 (43)
Anti-estrogen and aromatase inhibitor (sequential)	20 (3)	34 (4)
Aromatase inhibitor only	362 (44)	379 (47)
Non-anti-estrogen or aromatase inhibitor	3 (<1)	4 (<1)

Data are *N* (%), unless otherwise stated.

ER, estrogen receptor; IQR, interquartile range; PR, progesterone receptor.

[a]Because of rounding, not all percentages total to 100.

[b]Stratification factor collected from the interactive voice- and web-response system. For nodal status, the number of positive nodes was recorded at the time of initial diagnosis (for those who received adjuvant therapy) or surgery (for those who received neoadjuvant therapy). Patients with residual invasive disease in the breast but node negative or unknown nodal status in the axilla after neoadjuvant therapy were included under “1–3” positive nodes.

[c]Percentage is based on the number of patients with hormone receptor-positive disease. Tumors were assessed as being ER or PR positive based on local pathology laboratory cut-offs. There was no protocol specification as to whether a 1% or 10% threshold should be used.

[d]Proportion of patients who received neoadjuvant chemotherapy in the neratinib group was 25% and in the placebo group it was 27%.

Supplementary Table 3. Drug exposure by diarrhea grade (safety population)

	**Neratinib (*N* = 1408)**[a]	**Placebo (*N* = 1408)**
**Grade 1 or no diarrhea, *N***	**388**	**1291**
Median (IQR) treatment duration, months	11.7 (5.1–12.0)	11.8 (11.5–12.0)
Mean (SD) relative dose intensity, %	96 (10)	98 (5)
Dose reduction, *N* (%)	52 (13)	96 (7)
Dose hold, *N* (%)	154 (40)	554 (43)
**Grade 2 diarrhea, *N***	**458**	**94**
Median (IQR) treatment duration, months	11.7 (3.2–12.0)	11.9 (11.5–12.0)
Mean (SD) relative dose intensity, %	90 (15)	97 (5)
Dose reduction, *N* (%)	150 (33)	11 (12)
Dose hold, *N* (%)	278 (61)	55 (59)
**Grade 3 diarrhea, *N***	**561**	**23**
Median (IQR) treatment duration, months	11.5 (1.3–11.9)	11.6 (11.0–11.9)
Mean (SD) relative dose intensity, %	80 (21)	96 (7)
Dose reduction, *N* (%)	317 (57)	2 (22)
Dose hold, *N* (%)	417 (74)	14 (61)

IQR, interquartile range; SD, standard deviation.

[a]In the neratinib group, 1 patient with grade 4 diarrhea was excluded.

Supplementary Table 4. Blood chemistry results outside of normal range (safety population)

Parameter, *N* (%)	Normal range	Neratinib (*N* = 1408)	Placebo (*N* = 1408)
Albumin	35–50 g/L	*N* = 1384	*N* = 1389
High		86 (6)	104 (8)
Low		103 (7)	91 (7)
Blood urea nitrogen	3.6–7.1 mmol/L	*N* = 700	*N* = 703
High		237 (34)	221 (31)
Low		212 (30)	242 (34)
Calcium	2.25–2.62 mmol/L	*N* = 1405	*N* = 1407
High		52 (4)	72 (5)
Low		687 (49)	604 (43)
Creatinine	44–97 Umol/L	*N* = 1403	*N* = 1407
High		176 (13)	129 (9)
Low		62 (4)	100 (7)
Lactate dehydrogenase	105–333 U/L	*N* = 1394	*N* = 1400
High		348 (25)	370 (26)
Low		25 (2)	32 (2)
Magnesium	0.65–1.0 mmol/L	*N* = 1392	*N* = 1400
High		72 (5)	84 (6)
Low		100 (7)	71 (5)
Phosphate	0.97–1.45 mmol/L	*N* = 1386	*N* = 1398
High		161 (12)	196 (14)
Low		630 (46)	604 (43)
Potassium	3.5–5 mmol/L	*N* = 1406	*N* = 1407
High		158 (11)	176 (13)
Low		105 (8)	111 (8)
Sodium	136–145 mmol/L	*N* = 1407	*N* = 1407
High		146 (10)	150 (11)
Low		162 (12)	153 (11)

Supplementary Fig 1. ExteNET CONSORT flowchart

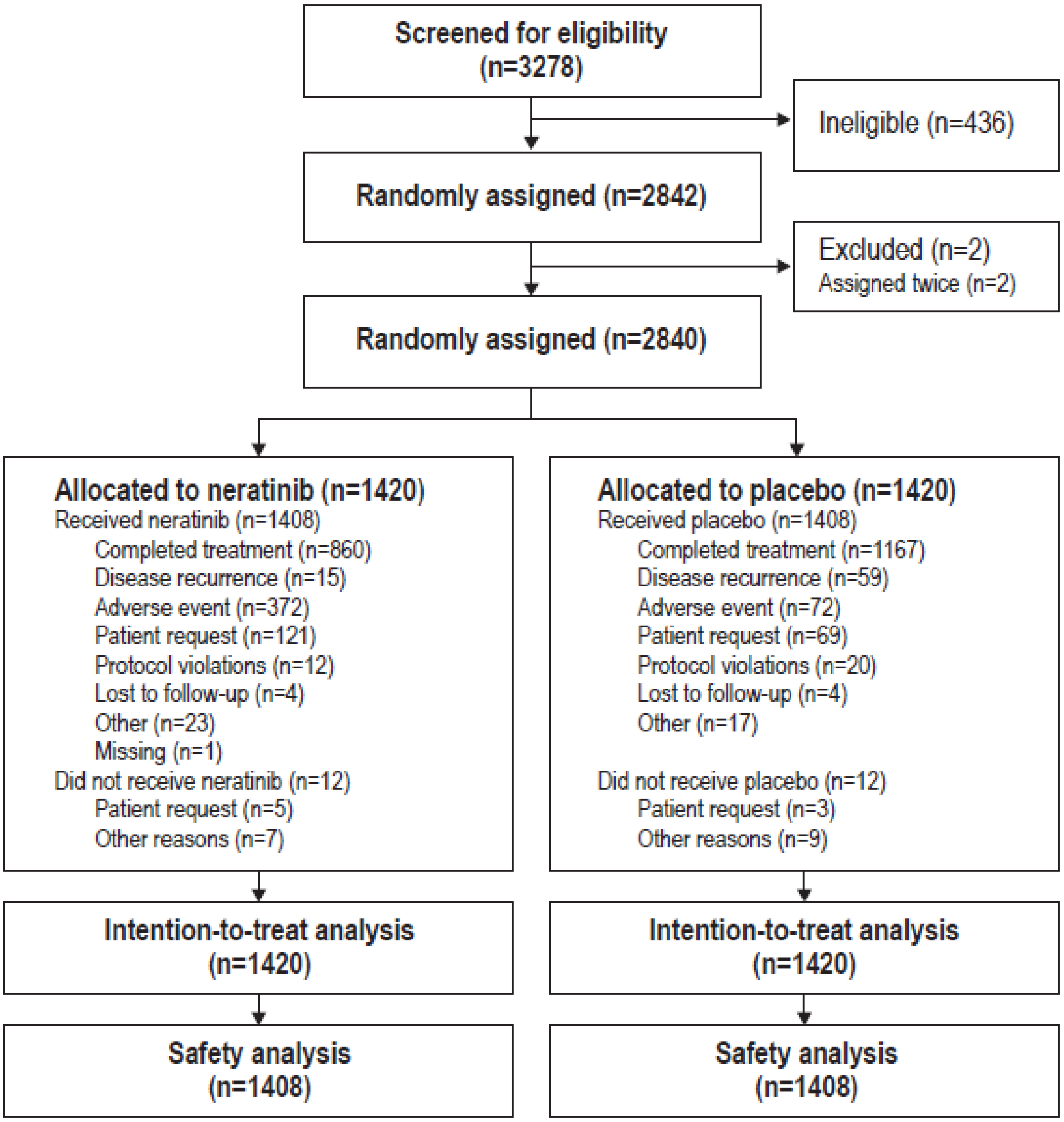

Supplementary Fig 2. Kaplan-Meier curves of invasive disease-free survival by worst grade of diarrhea experienced by patients in the neratinib group in the first 7 days (intention-to-treat population with a treatment duration >1 month)

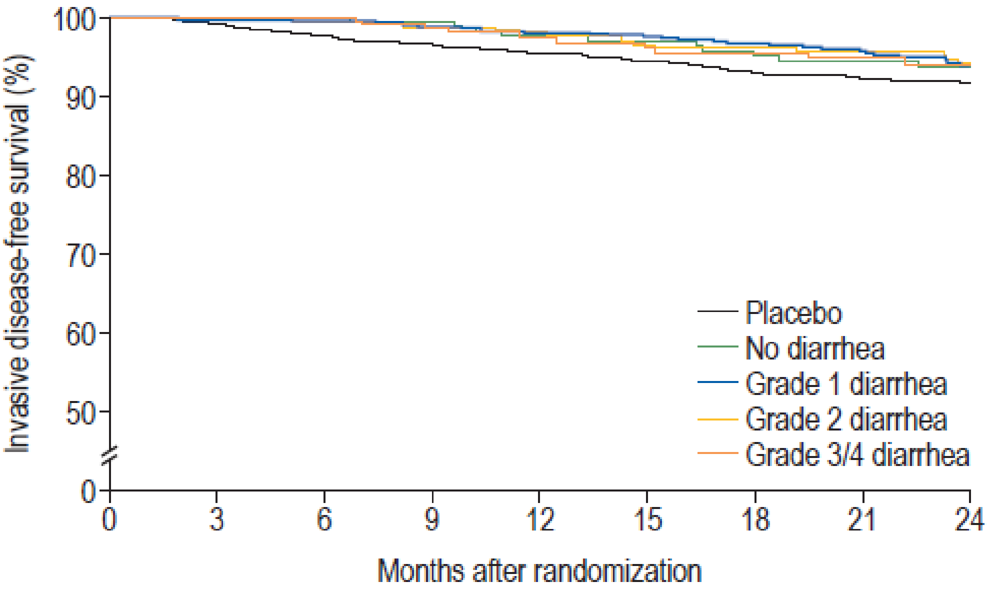

Placebo	1374	1355	1311	1279	1232	1197	1152	1080	697
No diarrhea	187	183	178	173	166	161	154	145	93
Grade 1 diarrhea	495	486	479	468	457	445	430	401	244
Grade 2 diarrhea	285	280	272	267	257	247	241	228	156
Grade 3/4 diarrhea	172	165	162	158	154	150	143	134	88

Invasive disease-free survival defined as the time from randomization to first occurrence of invasive ipsilateral tumor recurrence, invasive contralateral breast cancer, local/regional invasive recurrence, distant recurrence, or death from any cause.

Supplementary Fig 3. Mean (± standard deviation) Functional Assessment of Cancer Therapy – Breast (FACT-B) total scores over time by diarrhea grade for (A) neratinib and (B) placebo with last observation carried forward

A) Neratinib

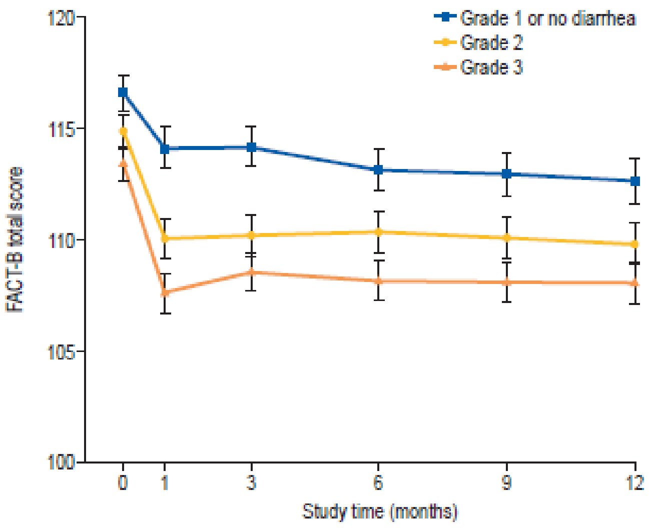

Number of Patients:

Grade 1 or no diarrhea	299	278	292	297	298	299
Grade 2	383	371	382	383	383	383
Grade 3	488	474	485	488	488	488

B) Placebo

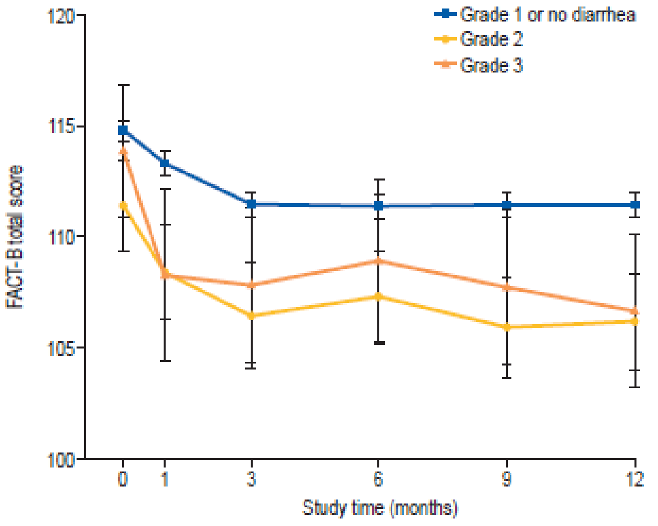

Number of Patients:

Grade 1 or no diarrhea	1126	1082	1118	1124	1124	1126
Grade 2	88	84	87	88	88	88
Grade 3	22	22	22	22	22	22

Minimally important difference: 7–8 points. A higher score indicates better quality of life.
